# Sex differences in hemodynamic responses and long-term survival to optimal medical therapy in patients with pulmonary arterial hypertension

**DOI:** 10.1007/s00380-018-1140-6

**Published:** 2018-02-13

**Authors:** Katsuya Kozu, Koichiro Sugimura, Tatsuo Aoki, Shunsuke Tatebe, Saori Yamamoto, Nobuhiro Yaoita, Toru Shimizu, Kotaro Nochioka, Haruka Sato, Ryo Konno, Kimio Satoh, Satoshi Miyata, Hiroaki Shimokawa

**Affiliations:** Department of Cardiovascular Medicine, Tohoku University Graduate School of Medicine, 1-1 Seiryo-machi, Aoba-ku, Sendai, 980-8574 Japan

**Keywords:** Pulmonary arterial hypertension, Sex difference, Right ventricular function, Pulmonary hemodynamics, Prognosis

## Abstract

It is widely known that the incidence of pulmonary arterial hypertension (PAH) is higher in female, whereas prognosis is poorer in male patients. However, sex differences in hemodynamic response to and long-term prognosis with PAH-targeted treatment in the modern era remain to be fully elucidated. We examined the long-term prognosis of 129 consecutive PAH patients (34 males and 95 females) diagnosed in our hospital from April 1999 to October 2014, and assessed hemodynamic changes in response to PAH-targeted therapy. Female patients had better 5-year survival compared with male patients (74.0 vs. 53.4%, *P* = 0.003); however, higher age quartiles in females were associated with poor outcome. Follow-up examination after medical treatment showed significant decreases in mean pulmonary arterial pressure (mPAP), pulmonary vascular resistance (PVR), and pulmonary arterial capacitance (PAC) in both sexes (both *P* < 0.05), whereas only females had a significant improvement in right ventricular end-diastolic pressure (RVEDP), right atrial pressure (RAP), cardiac index, and mixed venous oxygen saturation (SvO_2_) (all *P* < 0.05). Baseline age significantly correlated with the hemodynamic changes only in female patients; particularly, there were significant sex interactions in RVEDP and RAP (both *P* < 0.10). The multivariable analysis showed that SvO_2_ at baseline and mPAP and SvO_2_ at follow-up were significant prognostic factors in males, whereas the changes in mPAP, PVR, and PAC and use of endothelin-receptor antagonist in females. These results indicate that female PAH patients have better long-term prognosis than males, for which better improvements of right ventricular functions and hemodynamics may be involved.

## Introduction

Pulmonary arterial hypertension (PAH) is a disease characterized by progressive pulmonary vascular remodeling that increases pulmonary arterial pressure and finally leads to right heart failure and premature death [1]. In the past 2 decades, PAH-targeted medical therapy for major 3 pathways have been developed, including prostacyclin, endothelin-1 (ET-1), and nitric oxide (NO), with resultant marked improvement of survival of PAH patients [2–5]. Responses to the PAH-targeted treatment are heterogeneous, and epoprostenol, an intravenous prostacyclin analog, and macitentan, a dual endothelin-receptor antagonist, have only been shown to improve survival of PAH patients in randomized controlled trials [6–8]. Furthermore, it is widely known that the incidence of PAH is higher in females than in males, whereas long-term prognosis of PAH patients is poorer in males than in females [3, 9, 10]. This paradoxical phenomenon could be partially explained by the detrimental vs. protective effects of sex hormones in PAH patients; however, it remains to be examined what mechanism(s) is involved in such sex differences.

Responses of the right ventricle (RV) in response to increased hemodynamic load, such as right atrial pressure (RAP) and cardiac index (CI), are known to be significant prognostic factors of PAH [7, 11–13]. These clinical parameters of RV functions are also be poorer in male PAH patients at diagnosis [10, 14, 15]. However, sex differences in the relationship between hemodynamic parameters and long-term prognosis after PAH-specific therapy also remain to be elucidated. This point is important when developing personalized medicine by sex for each PAH patient. In the present study, we thus examined sex differences in the hemodynamic responses to and long-term prognosis with PAH-specific medical therapy in PAH patients.

## Methods

### Study population and treatment

The present study was approved by the Ethics Committee of Tohoku University Graduate School of Medicine (2012-1-301, 2016-1-582) and all patients provided written informed consent. From April 1999 to October 2014, we enrolled 129 consecutive patients with PAH who were diagnosed in our hospital. The diagnosis of PAH was made based on the established approaches, including physical examination, blood tests, echocardiography, pulmonary function test, chest X-ray, computed tomography (CT), ventilation-perfusion scanning, and right heart catheterization [16]. PAH subtype was classified based on the Nice classification [17]. PAH-specific drug therapy during the study period in Japan included oral and intravenous prostacyclin analogues since 1999, endothelin-receptor antagonists (ERAs) since 2005, and phosphodiesterase type-5 (PDE-5) inhibitors since 2008. These drugs were chosen by a physician in charge as a monotherapy or combination therapy. PAH-specific drugs were listed for the present analysis when the largest number of drugs were used during the follow-up. Baseline demographic data were collected from the medical records of each patient. Follow-up hemodynamic data were obtained at least 12 weeks after right heart catheterization at baseline in 97 out of 129 patients. Follow-up was completed in November 2016. The primary outcome was the composite end-point of all-cause death and lung transplantation [8, 18, 19].

### Statistical analysis

Continuous variables are expressed as the mean ± SD and categorical variables as the number (%). Means and percentages were compared using paired *t* test, Wilcoxon signed-rank test, *χ*^2^ test, or Fisher exact test, as appropriate. Event-free survival time was calculated from the date of diagnostic catheterization to the date of any cause of death, lung transplantation, or last follow-up. A Kaplan–Meier curve was used to estimate the overall event-free survival, and differences between survival curves were assessed using the log-rank test. Univariable and multivariable Cox proportional hazard models were used to estimate the hazard ratios and 95% confidence intervals. *P* values of < 0.05 were regarded to be statistically significant. All analyses were performed with JMP Pro 12.2.0 (Japanese version, SAS Institute Inc., Tokyo, Japan).

## Results

### Clinical characteristics of PAH patients

The number of patients with clinical subtypes of PAH was as follows; idiopathic/heritable PAH (IPAH/HPAH) in 45, connective tissue diseases (CTD) in 41, congenital heart disease (CHD) in 31, portal hypertension in 11, and drug- and toxin-induced in one. Clinical characteristics of the 129 patients are shown in Table 1. Mean age was 45 ± 18 years and 34 (26%) were male. Among them, 30 (23%) were treated with monotherapy, 84 (65%) with combination therapy with 2–3 PAH-specific drugs, and 40 (31%) with intravenous prostacyclin. During the mean observation period of 5.9 years, 43 (33%) patients died and 11 (9%) underwent lung transplantation.

**Table 1:** Sex differences in clinical characteristics, hemodynamics, and medical therapy in PAH patients

	Overall	Male	Female	*P* value
*N*	129	34	95	
Age (years)	45 ± 18	43 ± 20	45 ± 17	0.65
Time between baseline and follow-up (years)	1.2 ± 1.5	1.4 ± 2.0	1.1 ± 1.4	0.60
Mean follow-up duration (years)	5.9 ± 4.3	4.8 ± 3.6	6.3 ± 4.4	0.09
Subtype of PAH				
IPAH, *n* (%)	45 (35)	13 (38)	32 (34)	
Drug and toxin, *n* (%)	1 (1)	0 (0)	1 (1)	
CTD, *n* (%)	41 (32)	5 (15)	36 (38)	
Portal HT, *n* (%)	11 (9)	4 (12)	7 (7)	
CHD, *n* (%)	31 (24)	12 (35)	19 (20)	
WHO-FC III or IV, *n* (%)	52 (40)	13 (38)	39 (41)	0.84
BNP (pg/mL)	273 ± 389	210 ± 170	295 ± 440	0.96
Hemodynamics				
mPAP (mmHg)	50.6 ± 20.0	52.4 ± 20.0	50.0 ± 20.1	0.45
PAWP (mmHg)	8.5 ± 3.8	9.5 ± 3.8	8.2 ± 3.7	0.09
RVEDP (mmHg)	9.8 ± 4.6	10.1 ± 4.8	9.6 ± 4.5	0.60
RAP (mmHg)	6.8 ± 4.2	7.5 ± 4.1	6.5 ± 4.2	0.21
CI (L/min/m^2^)	2.79 ± 0.88	2.85 ± 0.96	2.76 ± 0.86	0.65
PVR (dyn/s/cm^5^)	933 ± 731	892 ± 727	948 ± 736	0.53
Heart rate (bpm)	79.8 ± 14.7	80.4 ± 14.1	79.6 ± 14.9	0.78
Pulmonary pulse pressure (mmHg)	44.2 ± 17.6	43.1 ± 17.9	44.7 ± 17.6	0.69
PAC (mL/mmHg)	1.52 ± 0.94	1.67 ± 0.96	1.46 ± 0.93	0.20
SvO_2_ (%)	67.7 ± 10.2	68.3 ± 11.8	67.4 ± 9.6	0.69
Medical therapy				
Epoprostenol, *n* (%)	40 (31)	8 (24)	32 (34)	0.39
Beraprost, *n* (%)	53 (41)	14 (41)	39 (41)	1.00
ERA, *n* (%)	83 (64)	22 (65)	61 (64)	1.00
PDE-5 inhibitor, *n* (%)	77 (60)	22 (65)	55 (58)	0.54
No PAH-targeted drug, *n* (%)	15 (12)	5 (15)	10 (11)	0.54
Monotherapy, *n* (%)	30 (23)	7 (21)	23 (24)	0.81
Double combination therapy, *n* (%)	29 (22)	7 (21)	22 (23)	0.82
Triple combination therapy, *n* (%)	55 (43)	15 (44)	40 (42)	0.84

Continuous variables are expressed as mean ± SD, *n* (%)

*BNP* brain natriuretic peptide, *CHD* congenital heart disease, *CI* cardiac index, *CTD* connective tissue diseases, *ERA* endothelin-receptor antagonist, *IPAH* idiopathic pulmonary arterial hypertension, *mPAP* mean pulmonary arterial pressure, *PAC* pulmonary arterial capacitance, *PAWP* pulmonary artery wedge pressure, *PDE-5* phosphodiesterase type-5, *Portal HT* portal hypertension, *PVR* pulmonary vascular resistance, *RAP* right atrial pressure, *RVEDP* right ventricular end-diastolic pressure, *SvO*_*2*_ mixed venous oxygen saturation, *WHO-FC* World Health Organization-functional class

### Long-term prognosis of PAH patients

**Fig. 1:**
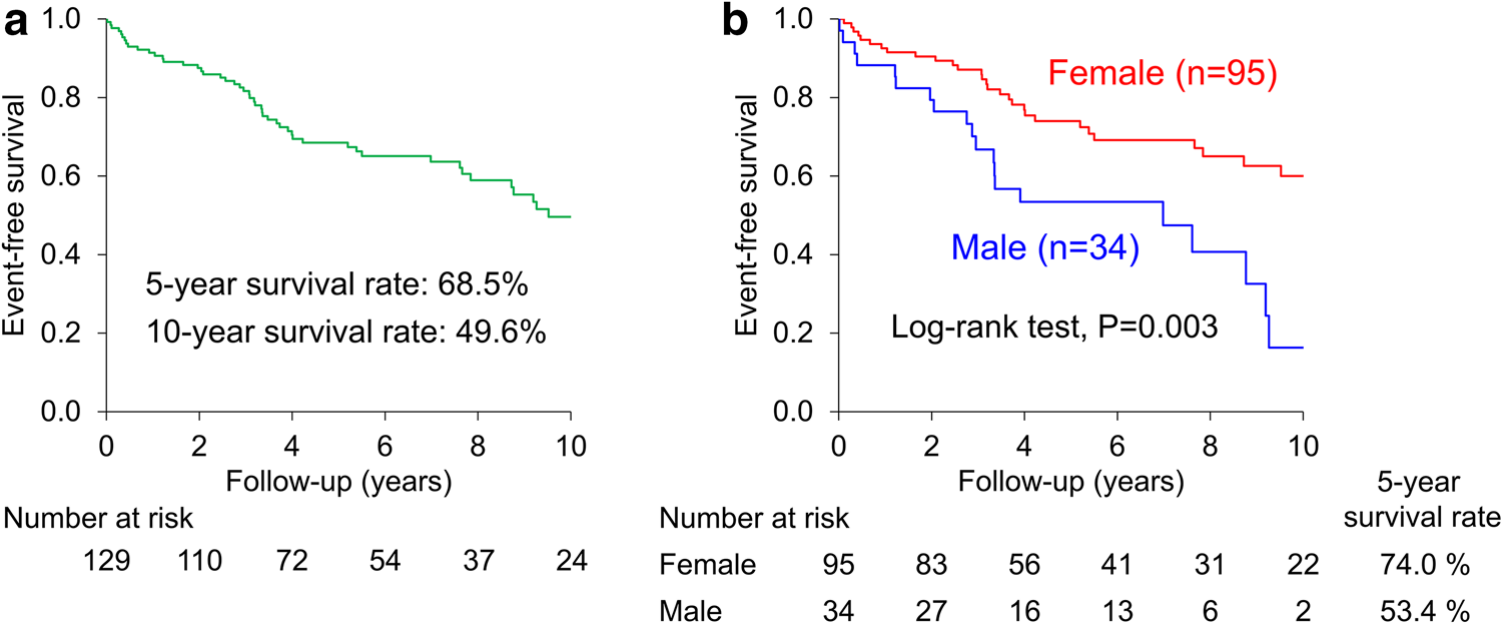
Long-term prognosis of PAH patients. **a** Event-free survival was 68.5% at 5 years and 49.6% at 10 years in all PAH patients. **b** Female patients had a better survival compared with male patients (*P* = 0.003)

Event-free survival in all PAH patients was 68.5% at 5 years and 49.6% at 10 years (Fig. 1a). Multivariable analysis at baseline showed that male sex, elderly age older than 60 years, World Health Organization-functional class (WHO-FC) III or IV, and higher mixed venous oxygen saturation (SvO_2_) at baseline were significant predictors for mortality (Table 2).

**Table 2:** Univariable and multivariable Cox proportional hazards model of PAH patients

Candidate variables	Univariable		Multivariable	
	HR (95% CI)	*P* value	HR (95% CI)	*P* value
Male	2.29 (1.28–4.00)	0.006	2.63 (1.41–4.80)	0.003
Age < 32 years	Reference		Reference	
32 ≤ age < 43 years	0.70 (0.31–1.57)	0.38	0.86 (0.36–2.01)	0.72
43 ≤ age < 60 years	1.13 (0.51–2.51)	0.76	2.04 (0.87–4.75)	0.10
Age ≥ 60 years	2.03 (0.97–4.35)	0.06	3.16 (1.45–7.07)	0.004
WHO-FC				
I or II	Reference		Reference	
III or IV	2.44 (1.42–4.24)	0.001	3.03 (1.71–5.47)	0.0001
BNP (per 100 pg/mL)	1.00 (0.91–1.07)	0.91		
mPAP (mmHg)	1.00 (0.99–1.02)	0.40		
PAWP (mmHg)	1.02 (0.94–1.09)	0.64		
RVEDP (mmHg)	1.06 (0.998–1.12)	0.06		
RAP (mmHg)	1.03 (0.97–1.08)	0.38		
CI (L/min/m^2^)	0.92 (0.66–1.27)	0.62		
PVR (per 100 dyn/s/cm^5^)	1.02 (0.98–1.05)	0.42		
PAC (mL/mmHg)	0.79 (0.54–1.09)	0.16		
SvO_2_ (%)	0.97 (0.94–0.998)	0.04	0.96 (0.93–0.99)	0.01

See Table 1 for abbreviations

### Sex differences in clinical characteristics and long-term prognosis of PAH patients

**Fig. 2:**
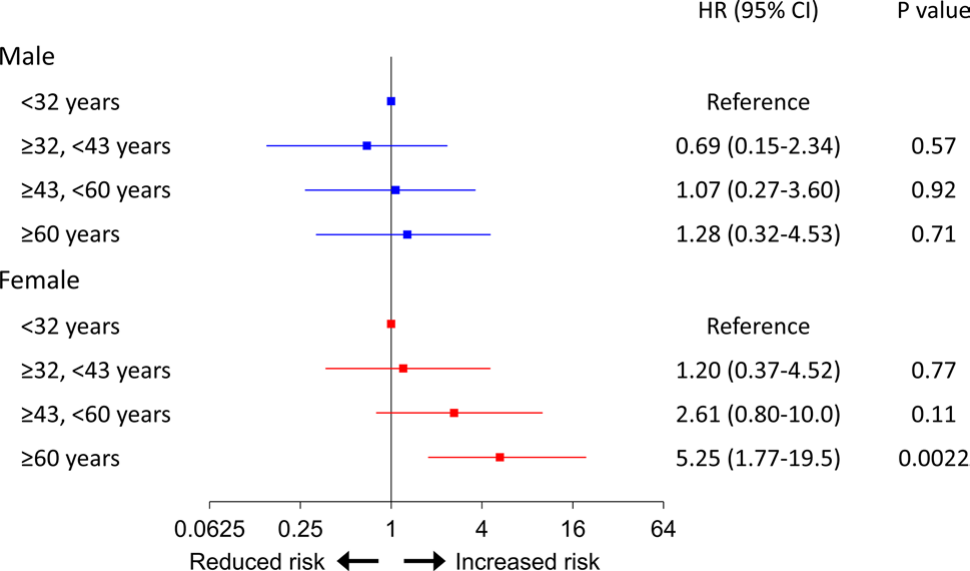
Multivariable Cox proportional hazard model of baseline age divided into 4 quartiles adjusted for WHO-FC. Elderly group was significantly associated with poor outcome in females but not in males

There were no significant sex differences in age, hemodynamic parameters, or severity of PH at baseline (Table 1). No sex difference was also noted for the use of PAH-specific drugs or the prevalence of combination therapy. However, female patients had better survival compared with male patients (5-year event-free survival rate, 74.0 vs. 53.4%, *P* = 0.003) (Fig. 1b). At baseline, when the patients were divided into 4 quartiles by age, elderly age (≥ 60 years) was significantly associated with poor outcome in the multivariable analysis adjusted for WHO-FC in females but not in males (Fig. 2).

### Hemodynamic changes in response to optimal medical therapy

Follow-up examination after medical therapy showed a significant decrease in mean pulmonary arterial pressure (mPAP) and pulmonary vascular resistance (PVR) and a significant increase in pulmonary arterial capacitance (PAC) in both sexes (both *P* < 0.05), whereas only female patients had significant decreases in right ventricular end-diastolic pressure (RVEDP) and right atrial pressure (RAP) and significant increases in cardiac index (CI) and SvO_2_ (Table 3). Importantly, a significant sex difference was noted in terms of change in RVEDP and tendency in that of RAP (Table 3).

**Table 3:** Sex differences in the hemodynamic changes after medical therapy in PAH patients

	Male		Female		*P* value for sex
	Change	*P* value between baseline and follow-up	Change	*P* value between baseline and follow-up	
mPAP (mmHg)	− 6.1 ± 9.0	0.004	− 7.1 ± 10.7	< 0.0001	0.70
RVEDP (mmHg)	0.4 ± 3.6	0.40	− 1.2 ± 4.4	0.008	0.06
RAP (mmHg)	− 0.1 ± 2.5	0.93	− 1.3 ± 3.9	0.007	0.11
CI (L/min/m^2^)	0.14 ± 0.99	0.11	0.29 ± 0.91	0.023	0.90
PVR (dyn/s/cm^5^)	− 320 ± 569	0.01	− 257 ± 454	< 0.0001	0.78
PAC (mL/mmHg)	0.43 ± 0.76	0.02	0.54 ± 0.72	< 0.0001	0.55
SvO_2_ (%)	1.1 ± 8.1	0.56	2.5 ± 8.8	0.027	0.52

See Table 1 for abbreviations

Baseline age significantly correlated with mPAP in male patients, and mPAP, RVEDP, and PVR in female patients; however, there were no significant sex interactions for the correlations (Table 4). In contrast, baseline age significantly correlated with the hemodynamic changes in mPAP, RAP, CI, PVR, and SvO_2_ only in female patients; particularly, there were significant sex interactions for the correlations between age and hemodynamic changes in RVEDP and RAP (Table 4).

**Table 4:** Sex differences in the correlation between baseline age and hemodynamics

	Male		Female		*P* value for interaction
	*R*	*P* value	*R*	*P* value	
Baseline					
mPAP (mmHg)	− 0.49	0.003	− 0.50	< 0.0001	0.63
RVEDP (mmHg)	− 0.19	0.28	− 0.32	0.002	0.39
RAP (mmHg)	− 0.13	0.46	− 0.18	0.08	0.69
CI (L/min/m^2^)	0.08	0.66	0.17	0.11	0.62
PVR (dyn/s/cm^5^)	− 0.30	0.10	− 0.36	0.001	0.54
PAC (mL/mmHg)	0.19	0.28	0.19	0.07	0.89
SvO_2_ (%)	− 0.03	0.86	0.19	0.07	0.26
Changes					
ΔmPAP (mmHg)	− 0.014	0.95	0.27	0.02	0.23
ΔRVEDP (mmHg)	− 0.26	0.28	0.22	0.06	0.08
ΔRAP (mmHg)	− 0.31	0.18	0.25	0.03	0.05
ΔCI (L/min/m^2^)	− 0.26	0.25	− 0.27	0.02	0.95
ΔPVR (dyn/s/cm^5^)	0.22	0.35	0.30	0.01	0.95
ΔPAC (mL/mmHg)	− 0.12	0.61	− 0.13	0.26	0.97
ΔSvO_2_ (%)	0.043	0.86	− 0.32	0.01	0.14

Δ indicates change in each hemodynamics. See Table 1 for abbreviations

### Prognostic effects of hemodynamics and PAH-specific medical therapy

The multivariable analysis adjusted for WHO-FC showed that SvO_2_ at baseline and mPAP and SvO_2_ at follow-up were significant prognostic factors in male patients, while the decrease in mPAP and PVR and the increase in PAC in female patients (Table 5). In addition, the use of ERA or PDE-5 inhibitors significantly correlated with a better prognosis in females but not in males (Table 5). Interestingly, a significant sex interaction was noted only for the use of ERA (Table 5). No significant correlation was noted between survival and the use of prostacyclin analogues, such as beraprost and epoprostenol, in both sexes (Table 5).

**Table 5:** Multivariable Cox proportional hazard models of the sex differences in hemodynamics and their changes in response to medical therapy, and PAH-targeted medical therapy adjusted for WHO-FC

	Male		Female		*P* value for interaction
	HR (95% CI)	*P* value	HR (95% CI)	*P* value	
Baseline					
mPAP per 10 mmHg	1.30 (0.995–1.726)	0.054	0.91 (0.74–1.10)	0.35	0.08
RAP per mmHg	1.01 (0.89–1.14)	0.82	0.98 (0.90–1.05)	0.58	0.64
CI per L/min/m^2^	0.87 (0.53–1.35)	0.54	1.19 (0.71–1.95)	0.51	0.47
PVR per 100 dyn/s/cm^5^	1.05 (0.98–1.12)	0.12	0.99 (0.92–1.04)	0.70	0.24
PAC per mL/mmHg	0.74 (0.42–1.18)	0.22	0.88 (0.51–1.40)	0.63	0.93
SvO_2_ per 10%	0.53 (0.30–0.90)	0.02	0.92 (0.63–1.39)	0.69	0.10
Follow-up					
mPAP per 10 mmHg	1.60 (1.04–2.48)	0.04	1.13 (0.85–1.47)	0.38	0.14
RAP per mmHg	1.14 (0.94–1.39)	0.18	1.08 (0.92–1.25)	0.33	0.60
CI per L/min/m^2^	1.20 (0.47–2.79)	0.69	0.65 (0.31–1.27)	0.21	0.16
PVR per 100 dyn/s/cm^5^	1.28 (0.97–1.70)	0.08	1.05 (0.96–1.15)	0.27	0.20
PAC per mL/mmHg	0.49 (0.13–1.29)	0.17	0.61 (0.29–1.11)	0.11	0.87
SvO_2_ per 10%	0.34 (0.12–0.86)	0.02	0.99 (0.59–1.76)	0.96	0.05
Changes					
Decrease in mPAP per 10 mmHg	0.61 (0.26–1.35)	0.24	0.55 (0.33–0.88)	0.013	0.89
Decrease in RAP per mmHg	0.97 (0.72–1.20)	0.80	0.98 (0.88–1.08)	0.66	0.64
Increase in CI per L/min/m^2^	1.07 (0.59–2.37)	0.83	0.68 (0.35–1.27)	0.24	0.22
Decrease in PVR per 100 dyn/s/cm^5^	1.10 (0.95–1.26)	0.19	0.88 (0.77–0.99)	0.034	0.02
Increase in PAC per mL/mmHg	0.67 (0.22–1.83)	0.44	0.29 (0.09–0.78)	0.013	0.20
Increase in SvO_2_ per 10%	0.62 (0.21–1.58)	0.32	1.04 (0.66–1.62)	0.85	0.33
Beraprost	2.03 (0.72–5.84)	0.18	1.09 (0.53–2.17)	0.82	0.30
Epoprostenol	0.78 (0.26–2.03)	0.62	0.72 (0.33–1.48)	0.37	0.94
ERA	2.02 (0.75–6.37)	0.17	0.42 (0.21–0.87)	0.02	0.02
PDE-5 inhibitor	0.73 (0.28–1.97)	0.52	0.45 (0.22–0.89)	0.02	0.65

See Table 1 for abbreviations

## Discussion

The novel findings of the present study are as follows: (1) event-free survival at 5 years in Japanese PAH patients was 68.5%, where female patients had superior survival compared with male patients, (2) aging was significantly associated with poor outcome in females but not in males, (3) in response to optimal medical therapy, several parameters, particularly RVEDP and RAP, were ameliorated in females but not in males, where significant sex interactions were noted in terms of the correlation between age and the changes in RVEDP and RAP, (4) significant prognostic factors were hemodynamics at baseline and follow-up in males but were hemodynamic changes in females, and (5) the uses of ERA and PDE-5 inhibitor were related to better prognosis in females but not in males. To the best of our knowledge, this is the first study demonstrating the sex differences in hemodynamic responses and long-term survival in response to optimal medical therapy in PAH patients.

### Sex differences in clinical characteristics in PAH

The prevalence of PAH is higher in females than in males in the general population [3, 9, 10], which was also the case in the present study. A number of experimental and clinical studies implicated the aggravating roles of estrogen in the pathogenesis of PAH, relating to tryptophan hydroxylase-1, 5-hydroxytryptamine, serotonin transporter, cytochrome P450 1B1, and mutations in bone morphogenetic protein receptor type 2 [20–23]. Through these pathways, estrogen accelerates cell proliferation and forming pulmonary artery lesions, leading to the development of PAH.

Although recent registry studies of IPAH patients showed that males had higher mPAP, PVR and RAP, and lower CI at diagnosis [9, 10, 15], no significant sex differences were noted in the present study. Since we enrolled patients of group 1 PAH with various etiologies, this may have resulted in the heterogeneity in hemodynamics as in the previous studies [24, 25].

### Sex differences in hemodynamic responses to optimal medical therapy in PAH

Increased RVEDP and RAP reflect RV overload or ischemia [14, 26, 27]. Hemodynamic and morphological parameters of RV functions are also important predictors of long-term survival of PAH patients [9, 18, 28]. Although it is generally known that female PAH patients tend to have more favorable RV function than males at diagnosis [3, 9, 10, 14], sex differences in RV functions in response to optimal medical therapy remain to be fully elucidated. The present study indicates that sex differences in hemodynamic responses to optimal medical therapy largely result from adaptation of RV function to pressure overload.

A number of studies showed that estrogens exert protective cardiovascular effects, which are dramatically reduced after menopause [29, 30]. Recent epidemiological studies also showed that higher estrogen levels were associated with better RV systolic function [31, 32]. In addition, experimental studies showed that estrogen exerted beneficial effects against PAH by preventing RV dysfunction and hypertrophy through inhibition of inflammation, fibrosis, and apoptotic signaling and improving mitochondrial function and RV contractility [33–37].

While estrogen has attracted much attention, androgens may also have essential parts in PAH. In a rodent model, androgens affect mal-adaptiveness for RV hypertrophy and fibrosis [38]. In addition, epidemiological studies showed that males had accelerated cardiomyocyte apoptosis, lower RV ejection fraction, and larger RV mass [39, 40], and that higher androgen levels were associated with poor RV functions [31].

In addition to the direct effects of sex hormones, PAH-targeted drugs may also have different effects for RV functions by sex. It was reported that PDE-5 inhibitor efficacy was estrogen-dependent in female mouse heart, which was mediated by enhanced cardiac synthesis of cyclic guanosine monophosphate (cGMP) through endothelial NO synthase and soluble guanylyl cyclase pathway [41]. This result supports our present finding of better RV responses after optimal medical therapy in female PAH patients compared with male patients.

### Sex differences in long-term prognosis with optical medical therapy in PAH

In the present study, although female PAH patients had worse hemodynamic conditions compared with male patients, they paradoxically had a better survival as in the previous studies [3, 9, 10]. However, sex differences in the prognostic factors in PAH patients remain to be examined. In the present study, aging was associated with worse prognosis only in female patients, suggesting negative effects of menopause. Moreover, a recent study demonstrated that there were sex differences in RV responses to medical therapy in PAH patients, which could explain a significant portion of the sex difference in survival in those patients [19, 42]. Taken together, these findings suggest that RV functions in response to optimal medical therapy are a key determinant of long-term prognosis of PAH patients. Indeed, in the present study, greater improvement in RV function-related parameters after optimal medication was associated with a better prognosis in females compared with males.

We also found that there are sex differences in the prognostic effects of PAH-targeted drugs, especially ERAs. It was previously reported that ERAs ameliorated 6-min walk distance (6MWD) and outcomes, especially in females [43]. This sex difference in clinical responses to ERAs may be related, at least in part, to higher levels of circulating ET-1 and greater vasoconstriction mediated by ET receptor type A in males [44, 45]. Sex differences in the effects of PDE-5 inhibitors have also been reported; male and premenopausal female patients were able to achieve clinically relevant responses in 6MWD and health-related quality of life [46, 47]. These findings may be explained by sexual dimorphism in NO metabolism, as urinary ^15^N nitrate excretion, which reflects total NO biosynthesis, was significantly higher in females compared with males, suggesting that males may benefit more from the NO pathway and PDE-5 inhibitors than females [48]. In the present study, no significant sex interaction was noted for PDE-5 inhibitors use as in the previous study [47]. This is probably because medication is effective for both sexes in different ways. Further studies are needed to explore the mechanisms of the sex differences in clinical responses to PAH-targeted drugs.

### Limitations

Several limitations should be mentioned for the present study. First, the present study is a single center one with possible selection bias. Event-free survival of PAH patients in the present study was poor compared with other 2 recent studies with Japanese IPAH/HPAH patients [42, 49]. This difference in mortality rate may be due to that in intravenous prostacyclin use (present study, 64% vs. Ogawa et al. 79%) [42] and that in enrollment period (present study, 1999–2014 vs. Tamura et al., 2008–2013) [49]. Second, in the present study, we enrolled all patients with Group 1 PAH; in particular, there was a relatively large number of CTD-PAH in female patients. However, event-free survival in systemic sclerosis-associated PAH (4 males, 7 females) showed no sex difference in the present study (log-rank test, *P* = 0.38) as in the previous study [50]. Even if we excluded patients with CTD-PAH, female patients showed a better prognosis (data not shown). Thus, we consider that the present findings could be generalizable to all forms of PAH. Third, there were 32 (25%) cases without follow-up right heart catheterization in the present study. However, among the patients without follow-up catheterization (13 males and 19 females), there was no sex difference in the long-term prognosis (log-rank test, *P* = 0.63). Finally, it is possible that long-term survivors had more chance to be treated with newly approved drugs during the follow-up period. Thus, further prospective studies are needed to examine the efficacy of the combination therapy of PAH-targeted drugs.

## Conclusions

In the present study, we were able to demonstrate that female PAH patients, particularly in younger patients, have better prognosis, for which a better improvement of RV functions in response to PAH-specific medical therapy may be involved. Further development of individual treatment may ameliorate long-term prognosis of PAH patients.
